# Prognostic impact of age on outcomes of hepatic decompensation in patients with compensated cirrhosis (CHESS2102): an international, multicenter cohort study

**DOI:** 10.1002/mco2.781

**Published:** 2024-11-03

**Authors:** Shanghao Liu, Jia Li, Yujun Wong, Hyung Joon Yim, Masashi Hirooka, Hirayuki Enomoto, Qing Xie, Erhei Dai, Amr Shaaban Hanafy, Zhujun Cao, Lili Zhao, Kok Ban Teh, Tae Hyung Kim, Young Kul Jung, Yohei Koizumi, Yoichi Hiasa, Takashi Nishimura, Hiroko Iijima, Qingyi Tian, Xinru Guo, Yansheng Jia, Jinfang Sun, Chuan Liu, Xiaolong Qi

**Affiliations:** ^1^ Liver Disease Center of Integrated Traditional Chinese and Western Medicine Department of Radiology Zhongda Hospital Medical School Southeast University Nurturing Center of Jiangsu Province for State Laboratory of AI Imaging & Interventional Radiology (Southeast University) Nanjing China; ^2^ Basic Medicine Research and Innovation Center of Ministry of Education Zhongda Hospital, Southeast University State Key Laboratory of Digital Medical Engineering Nanjing China; ^3^ Department of Gastroenterology and Hepatology Tianjin Second People's Hospital Tianjin China; ^4^ Department of Gastroenterology & Hepatology Changi General Hospital SingHealth Singapore Singapore; ^5^ Duke‐NUS Medical School Singapore Singapore; ^6^ Division of Gastroenterology and Hepatology Korea University Ansan Hospital Ansan Gyeonggi Republic of Korea; ^7^ Department of Gastroenterology and Metabology Ehime University Graduate School of Medicine Ehime Japan; ^8^ Division of Hepatobiliary and Pancreatic Diseases Department of Gastroenterology Hyogo Medical University Nishinomiya Japan; ^9^ Department of Infectious Disease Ruijin Hospital Shanghai Jiao Tong University School of Medicine Shanghai China; ^10^ Division of Liver Diseases The Fifth Hospital of Shijiazhuang North China University of Science and Technology Shijiazhuang China; ^11^ Division of Gastroenterology Hepatology and Endoscopy Internal Medicine Zagazig University Faculty of Medicine Zagazig Egypt; ^12^ Division of Gastroenterology and Hepatology Hallym University Sacred Heart Hospital Anyang Gyeonggi Republic of Korea; ^13^ Department of Internal Medicine Division of Gastroenterology and Hepatology Hyogo Medical University Nishinomiya Japan; ^14^ Ultrasound Imaging Center Hyogo Medical University Nishinomiya Japan; ^15^ Department of Epidemiology and Biostatistics School of Public Health Southeast University Nanjing China; ^16^ Key Laboratory of Environmental Medicine Engineering Ministry of Education School of Public Health Southeast University Nanjing China

**Keywords:** age, Baveno criteria, compensated cirrhosis, hepatic decompensation

## Abstract

Baveno VII criteria (B7C) and Baveno VI criteria (B6C) have been widely used to estimate the risk of hepatic decompensation. However, the impact of age on these criteria warrants further investigation. The international, multicenter cohort study included 1138 patients with compensated cirrhosis (median follow‐up of 40.6 months), aiming to evaluate the value of age in predicting hepatic decompensation. We identified age as an independent predictor of hepatic decompensation, with 60 years determined as the optimal cut‐off value. The occurrence of decompensation was 18.7% and 6.7% in the older (age ≥60 years) and younger (age <60 years) groups, respectively (*p* < 0.001). We subsequently integrated age into the existing Baveno criteria. In patients not meeting Baveno criteria (defined as not meeting B6C or B7C), the older group exhibited a significantly elevated risk of decompensation compared to the younger group (*p* < 0.05). However, no significant difference was observed between the older and younger groups in patients meeting Baveno criteria (*p* > 0.05). In conclusion, our study demonstrated that integrating age into the Baveno criteria could enhance the assessment of hepatic decompensation. Age should be considered before discharging patients with compensated cirrhosis from the surveillance of hepatic decompensation.

## Graphical abstract

Graphical Abstract: In this international multicenter cohort study, our study showed that integrating age into the Baveno criteria could enhance the assessment of hepatic decompensation. The older patients (age ≥60 years) who do not meet Baveno criteria have the highest risk of decompensation. Here, age should be considered before discharging patients with compensated cirrhosis from the surveillance of hepatic decompensation.

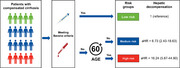

## 1 INTRODUCTION

Cirrhosis, characterized by diffuse liver fibrosis with nodular regeneration, stands as one of the leading global burdens of disease, resulting in more than one million deaths annually.^1^ In the United States alone, liver‐related expenses soared to $32.5 billion in 2016, with hospitalizations or emergency care comprising the majority of this expenditure.^2^ The progression of this disease begins with a prolonged asymptomatic phase known as compensated cirrhosis, eventually advancing to a symptomatic phase referred to as decompensated cirrhosis.^3^ The shift from compensated to decompensated cirrhosis happens at an annual rate of 5%–7%, significantly cutting median survival from 12 years to 2 years.^4^, ^5^ Recent findings from the Global Burden of Disease Study revealed that the global prevalence of cirrhosis nearly doubled between 1990 and 2017, with more than 90% of cases classified as compensated cirrhosis.^6^ Therefore, it is imperative to improve the management strategies for compensated cirrhosis and to accurately identify those individuals who are at an elevated risk of hepatic decompensation, as this will ultimately enhance their prognosis.

Portal hypertension serves as a major driver of hepatic decompensated. Although hepatic venous pressure gradient (HVPG) remains the gold standard for assessing portal hypertension, its invasive and costly nature limits widespread use.^7^ In recent years, several noninvasive methods have been proposed to evaluate portal hypertension and to predict the risk of decompensation.^8^, ^9^, ^10^ Among these, the Baveno VII criteria (B7C, platelet count [PLT] ≥150 × 10^9^/L and liver stiffness measurement [LSM] ≤15 kiloPascals [kPa]) and the Baveno VI criteria (B6C, PLT >150 × 10^9^/L and LSM <20 kPa) proposed by the Baveno workshop are the most widely recognized and validated.^4^, ^9^, ^11^

Over the last 50 years, along with socioeconomic development, the proportion of the aging population has substantially increased due to a noticeable decline in the fertility rate and a significant increase in the average life expectancy in most regions worldwide.^12^ As reported by the World Health Organization (WHO), the number of individuals aged 60 and older was estimated to be 962 million globally, accounting for 13% of the global population in 2017. By 2050, 20% of the global population is expected to be 60 years or older.^12^, ^13^ In patients with chronic liver disease, advanced age can lead to a poorer prognosis.^9^, ^14^, ^15^, ^16^ A systematic review of 118 studies indicated that age, as an essential and potent predictor of death, must be included in any prognostic study of cirrhosis, independent of disease stage.^5^ The latest Baveno VII consensus suggests establishing whether age impacts the performance of noninvasive tests for portal hypertension.^9^ Nonetheless, few studies have investigated the value of age in predicting hepatic decompensation in patients with compensated cirrhosis. This knowledge gap may have led to the fact that, to date, age has still not been included in risk stratification systems for these patients.

The study aimed to explore the value of age in predicting the first hepatic decompensation. Additionally, we endeavor to integrate age into the Baveno criteria to improve risk stratification for patients with compensated cirrhosis.

## 2 RESULTS

### 2.1 Baseline characteristics of included participants

**FIGURE 1:**
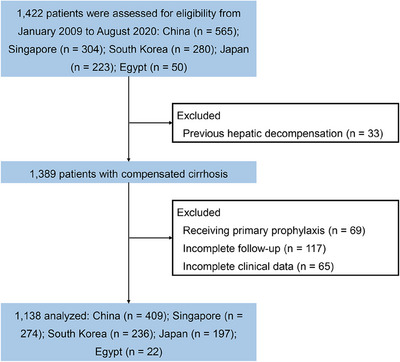
Flowchart of the study population.

From January 2009 to August 2020, a total of 1422 individuals from 5 countries were assessed for eligibility: China (*n* = 565), Singapore (*n* = 304), South Korea (*n* = 280), Japan (*n* = 223), and Egypt (*n* = 50). In accordance with the exclusion criteria, 1138 patients with compensated cirrhosis were included in the final analysis. The flowchart of patients is depicted in Figure 1. The mean age was 54.1 years (standard deviation [SD] 11.9), and 65.4% (*n* = 744) were male. The predominant etiology is viral hepatitis (*n* = 852, 74.9%), followed by nonalcoholic steatohepatitis (*n* = 106, 9.3%) and alcohol‐related liver disease (*n* = 64, 5.6%). The mean PLT was 141.5 × 10^9^/L, and the mean LSM was 19.6 (SD 13.5) kPa. A total of 118 patients (10.4%) developed hepatic decompensation during a median follow‐up of 40.6 months (interquartile range [IQR] 39.1–42.0). Patients were categorized into two groups based on whether hepatic decompensation occurred at the end of the follow‐up. Patients experiencing hepatic decompensation were older and had significantly higher baseline LSM, lower PLT, and worse liver function compared to those without the development of hepatic decompensation. Additional baseline characteristics are detailed in Table 1.

**TABLE 1:** Baseline characteristics.

Characteristic	Not hepatic decompensation (*n* = 1020)	Hepatic decompensation (*n* = 118)	*p* value
Age, years	53.3 (11.8)	60.7 (11.4)	<0.001
Male, *n* (%)	678 (66.5)	66 (55.9)	0.03
Etiology, *n* (%)			<0.001
Viral hepatitis	795 (77.9)	57 (48.3)	
NASH	88 (8.6)	18 (15.3)	
Alcohol	43 (4.2)	21 (17.8)	
Other^a^	94 (9.2)	22 (18.6)	
Body mass index, kg/m^2^	24.9 (4.1)	25.1 (4.7)	0.717
Creatinine, μmol/L	79.7 (73.3)	71.1 (36.9)	0.210
Total bilirubin, μmol/L	18.7 (15.7)	25.7 (24.9)	<0.001
Albumin, g/L	41.6 (4.9)	36.6 (5.7)	<0.001
LSM, kPa	18.0 (11.6)	33.9 (19.5)	<0.001
PLT, ×10^9^/L	147.2 (66.7)	92.1 (39.5)	<0.001
Varices, *n* (%)	308 (30.2)	90 (76.3)	<0.001
International normalized ratio	1.1 (0.1)	1.2 (0.2)	<0.001
MELD	8.4 (2.6)	10.3 (3.5)	<0.001
Child‐Pugh class, *n* (%)			0.004
A	986 (96.7)	107 (90.7)	
B	34 (3.3)	11 (9.3)	
Not meeting Baveno VI criteria, *n* (%)	678 (66.5)	114 (96.6)	<0.001
Not meeting Baveno VII criteria, *n* (%)	745 (73.0)	113 (95.8)	<0.001
Follow‐up, months	40.9 (27.9–54.5)	25.3 (13.6–37.6)	<0.001

*Note*: Data are *n* (%), mean (standard deviation), or medium (interquartile range).

Abbreviations: LSM, liver stiffness measurement; MELD, model of end‐stage liver disease; NASH, nonalcoholic steatohepatitis; PLT, platelet count.

^a^ Other etiologies included autoimmune hepatitis, primary biliary cholangitis, primary sclerosing cholangitis, drug‐induced liver injury, and cryptogenic cirrhosis; Baveno VII criteria: PLT ≥150 × 10^9^/L and LSM ≤15 kPa; Baveno VI criteria: PLT > 150 × 10^9^/L and LSM < 20 kPa.

### 2.2 Association between age and hepatic decompensation

**FIGURE 2:**
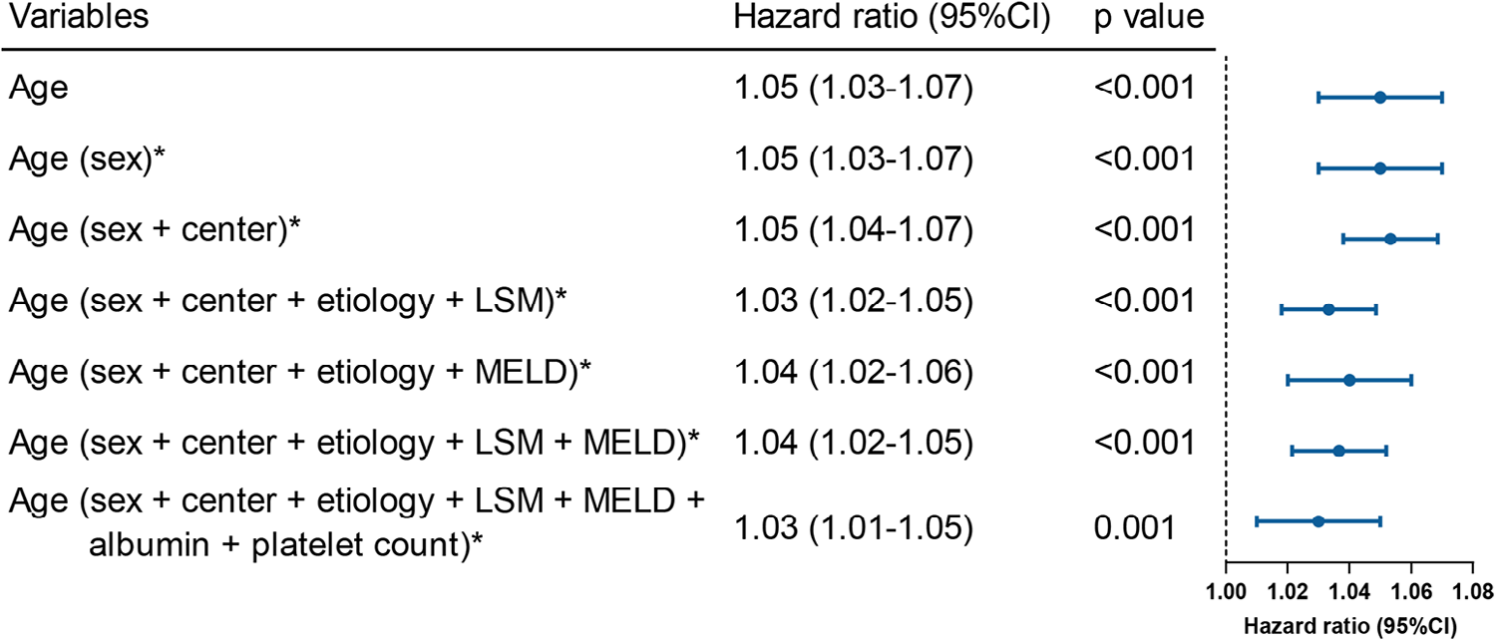
Multivariable Cox regression analysis of age as a predictor of hepatic decompensation. This forest plot displays the results of a multivariable Cox regression analysis. CI, confidence interval; LSM, liver stiffness measurement; MELD, model of end‐stage liver disease. *Age corrected for covariates in brackets.

The restricted cubic spline analysis showed a progressive increase in the risk of hepatic decompensation with advancing age, with a nonsignificant result for the nonlinearity test (*p* = 0.457), indicating a linear relationship (Figure S1A). Furthermore, age was significantly related to the occurrence of hepatic decompensation in univariate regression analysis (hazard ratio [HR] = 1.05 [95% CI: 1.03–1.07], *p* < 0.001). In multivariate regression analysis (Figure 2), age persisted as a significant predictor of hepatic decompensation after adjusting for sex and center (adjusted HR [aHR] = 1.05 [95% CI 1.04–1.07], *p* < 0.001). This association remained robust despite further balancing for etiology, LSM, model of end‐stage liver disease (MELD) score, albumin, and PLT (aHR = 1.03 [95% CI 1.01–1.05], *p* = 0.001). By employing maximally selected rank statistics, the optimal threshold for risk stratification was identified as 60 years of age (Figure S1B). Therefore, patients were divided into the younger group (age <60 years) and older group (age ≥60 years). Compared to the younger group, the older group had significantly higher LSM and MELD score, and a higher proportion of varices (Table 2). The follow‐up times were similar between the older and the younger groups (40.8 vs. 39.0 months, *p* = 0.764). By the end of follow‐up, the occurrence of decompensation was 18.7% (*n* = 65/347) and 6.7% (*n* = 53/791) in the older and younger groups, respectively, (*p* < 0.001).

**TABLE 2:** Clinical characteristics in the younger and older patients.

Characteristic	The younger	The older	*p* value
Age, years	48.0 (8.3)	67.8 (6.1)	<0.001
Male, *n* (%)	582 (73.6)	162 (46.7)	<0.001
Etiology, *n* (%)			<0.001
Viral hepatitis	655 (82.8)	197 (56.8)	
NASH	41 (5.2)	65 (18.7)	
Alcohol	33 (4.2)	31 (8.9)	
Other^a^	62 (7.8)	54 (15.6)	
Body mass index, kg/m^2^	25.1 (3.9)	24.6 (4.7)	0.067
Creatinine, μmol/L	76.5 (41.5)	84.0 (111.0)	0.098
Total bilirubin, μmol/L	19.7 (18.1)	18.7 (14.3)	0.354
Albumin, g/L	41.9 (5.0)	39.3 (5.4)	<0.001
LSM, kPa	18.9 (13.0)	21.4 (14.5)	0.003
PLT, ×10^9^/L	148.3 (69.0)	126.0 (57.7)	<0.001
Varices, *n* (%)	251 (31.7)	147 (42.4)	<0.001
International normalized ratio	1.1 (0.1)	1.1 (0.1)	0.297
MELD	8.5 (2.6)	9.0 (3.2)	0.007
Child‐Pugh class, *n* (%)			0.557
A	762 (96.3)	331 (95.4)	
B	29 (3.7)	16 (4.6)	
Meeting Baveno VI criteria, *n* (%)	278 (35.1)	68 (19.6)	<0.001
Meeting Baveno VII criteria, *n* (%)	228 (28.8)	52 (15.0)	<0.001
Hepatic decompensation, *n* (%)	53 (6.7)	65 (18.7)	<0.001
Follow‐up, months	39.0 (25.7–52.6)	40.8 (26.8–54.4)	0.764

*Note*: Data are *n* (%), mean (standard deviation), or medium (interquartile range).

Abbreviations: LSM, liver stiffness measurement; MELD, model of end‐stage liver disease; NASH, nonalcoholic steatohepatitis.

^a^ Other etiologies included autoimmune hepatitis, primary biliary cholangitis, primary sclerosing cholangitis, drug‐induced liver injury, and cryptogenic cirrhosis; Baveno VII criteria: PLT ≥150 × 10^9^/L and LSM ≤15 kPa; Baveno VI criteria: PLT > 150 × 10^9^/L and LSM < 20 kPa.

### 2.3 Risk stratification of hepatic decompensation based on Baveno criteria and age groups

**FIGURE 3:**
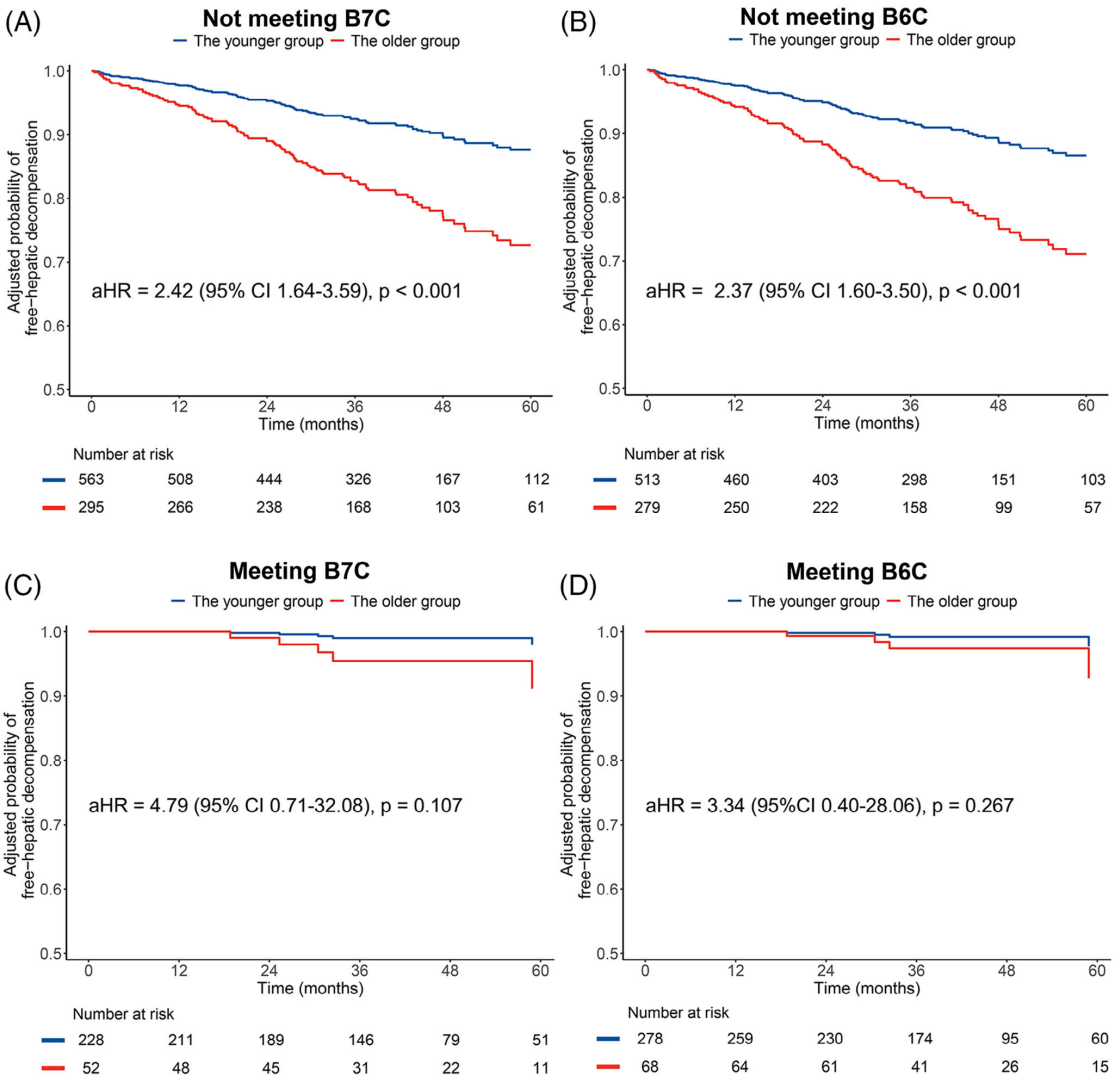
The adjusted probability of free‐hepatic decompensation between the older and younger groups in patients not meeting B7C (A) or B6C (B), and those meeting B7C (C) or B6C (D). Covariates included sex (female [reference] vs. male) and country (other countries [reference] vs. China). The adjusted curves were plotted using the direct standardization method by “adjustedCurves” package. aHR, adjusted hazard ratio; B6C, Baveno VI criteria; B7C, Baveno VII criteria.

In the total cohort, patients not meeting the B7C or B6C criteria had a higher risk of developing decompensation compared to those who met the criteria (both log‐rank tests: *p* < 0.001). The estimated 3‐year cumulative incidence of decompensation in patients meeting B7C or B6C was 1.9% and 1.1%, respectively, whereas in those not meeting the criteria, it was 10.9% and 11.9%, respectively. The risk of hepatic decompensation was significantly higher in the older group compared to the younger group among patients not meeting B7C (aHR = 2.42 [95% CI 1.64–3.59], *p* < 0.001) and B6C (aHR = 2.37 [95% CI 1.60–3.50], *p* < 0.001) after adjusting the covariates of sex and center (Figure 3A,B). Conversely, among patients who met B7C and B6C, there was no significant difference in the risk of hepatic decompensation between the older and younger groups, with aHRs of 4.79 (95% CI 0.71–32.08, *p* = 0.107) and 3.34 (95% CI 0.40–28.06, *p* = 0.267), respectively (Figure 3C,D). Independent subgroup analyses conducted in China, and other countries showed similar findings (Table S1). Consistent with the results from the overall cohort, we found no significant difference in the incidence rate of decompensation between older and younger patients in those meeting B7C and B6C groups; however, among those not meeting B7C and B6C criteria, there were significant differences between the older and younger groups.

**FIGURE 4:**
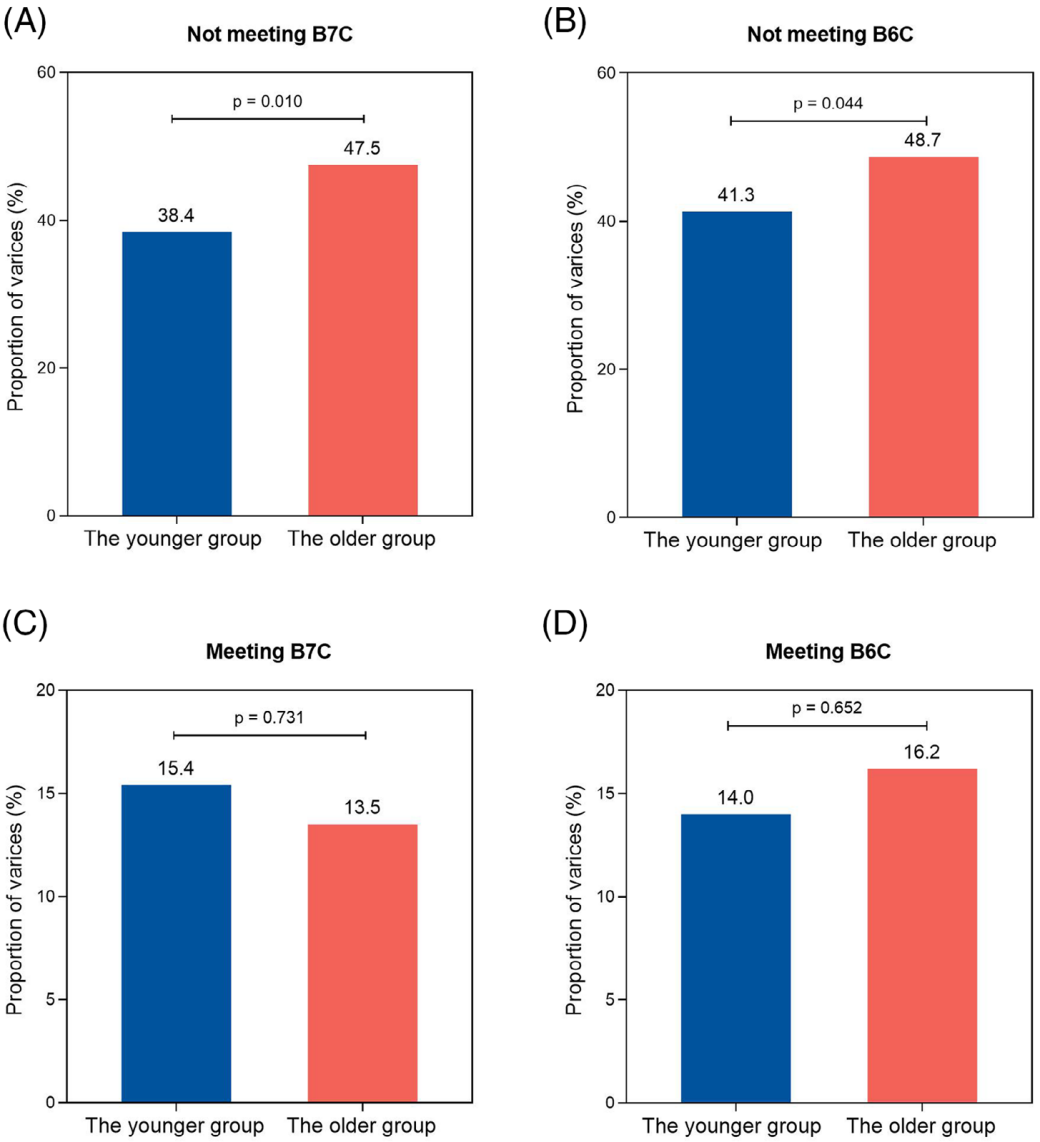
The distribution of varices between the older and younger groups in patients not meeting B7C (A) or B6C (B), and those meeting B7C (C) or B6C (D). The statistical significance of the differences was evaluated using the chi‐squared test. B6C, Baveno VI criteria; B7C, Baveno VII criteria.

Furthermore, among individuals not meeting B7C criteria, the proportion of varices was 47.5% (*n* = 140/295) in the older group and 38.4% (*n* = 216/563) in the younger group (*p* = 0.010, Figure 4A). Similarly, in those not meeting B6 criteria, the proportion of varices was 48.7% (*n* = 136/279) in the older group and 41.3% (*n* = 212/513) in the younger group (*p* = 0.044, Figure 4B). However, no significant difference in the proportion of varices between the older and younger groups was observed among individuals meeting B7C or B6C criteria (both *p* > 0.05, Figure 4C,D).

### 2.4 Incorporating age into the Baveno criteria

**FIGURE 5:**
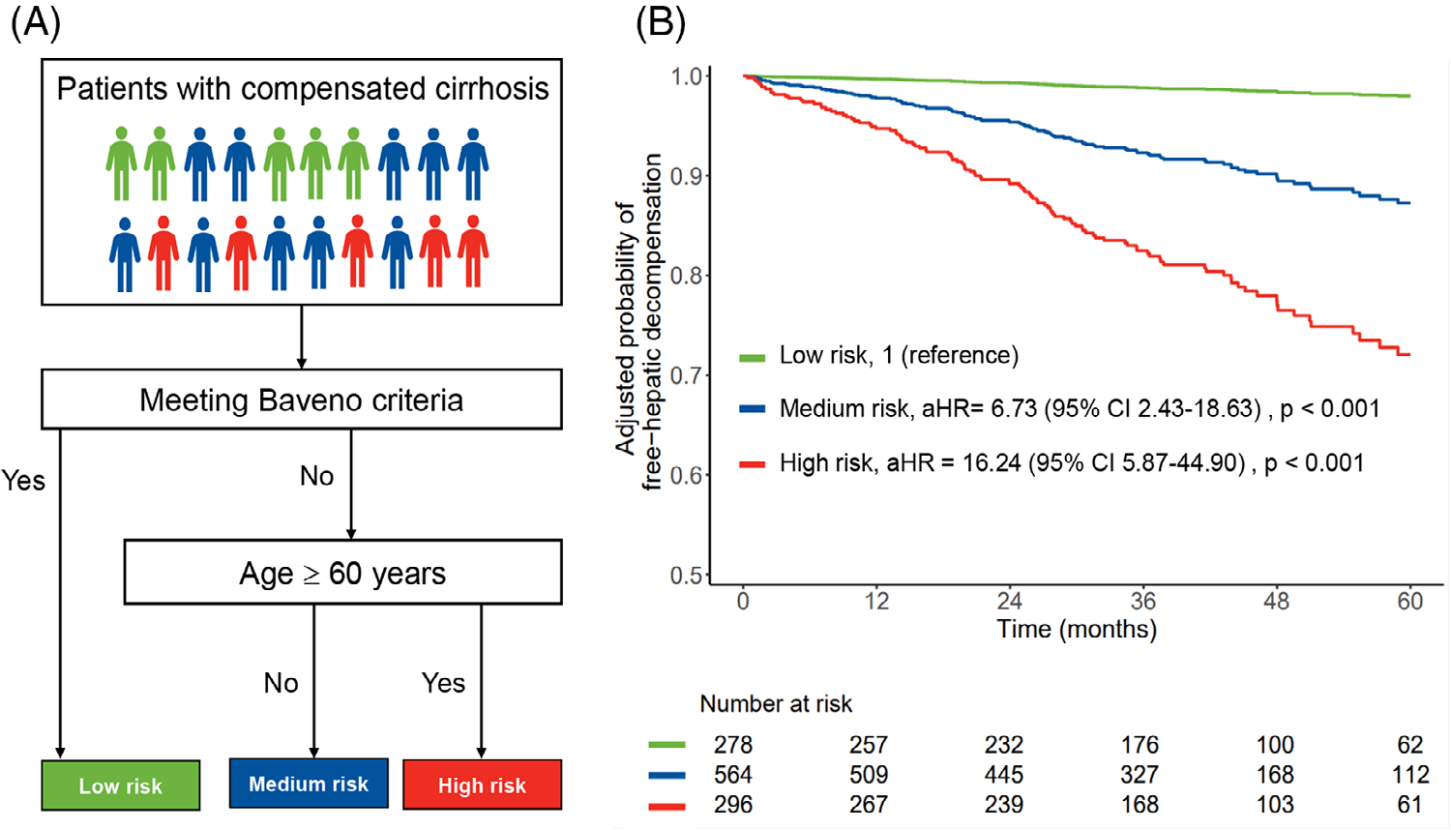
The pathway of Baveno criteria in combination with age groups to stratify the risk of hepatic decompensation (A) and the adjusted probability of free‐hepatic decompensation in different risk groups (B). Meeting the Baveno criteria is defined as satisfying the Baveno VII criteria and Baveno VI criteria simultaneously. Otherwise, it is considered not to meet the Baveno criteria. Covariates included sex (female [reference] vs. male) and country (other countries [reference] vs. China). The adjusted curves were plotted using the direct standardization method by “adjustedCurves” package.

We incorporated age into the Baveno criteria (Figure 5A). According to the clinical pathway, individuals who fulfilled the Baveno criteria were classified into the low‐risk group. Conversely, those who did not meet the Baveno criteria and were under 60 years old were placed in the medium‐risk group. Finally, individuals who failed to meet the Baveno criteria and were 60 years old or older were assigned to the high‐risk group.

The proportions of varices among the low‐, medium‐, and high‐risk groups were observed to be 15.1% (*n* = 42/278), 38.3% (*n* = 216/564), and 47.3% (*n* = 140/296), respectively. After adjusting for sex and center, the risk of hepatic decompensation was 6.73‐fold (aHR, 95% CI 2.43–18.63, *p* < 0.001) and 16.24‐fold (aHR, 95% CI 5.87–44.90, *p* < 0.001) higher in the medium‐ and high‐risk groups, respectively, compared with the low‐risk group (Figure 5B). Furthermore, a comparison between the medium‐ and high‐risk groups revealed that the latter exhibited a markedly increased risk of decompensation, with an aHR of 2.45 (95% CI 1.66–3.62, *p* < 0.001). By the end of follow‐up, the proportion of hepatic decompensation occurring in the low‐, medium‐, and high‐risk groups was 1.4% (*n* = 4/278), 9.0% (*n* = 51/564), and 21.3% (*n* = 63/296), respectively (*p* < 0.001).

## 3 DISCUSSION

In the international, multicenter cohort study, we identified age as an independent predictor of hepatic decompensation in patients with compensated cirrhosis. Importantly, our findings revealed that age exerted distinct prognostic implications at different stages of compensated cirrhosis. Specifically, older patients who did not satisfy the Baveno criteria exhibited a significantly elevated risk of hepatic decompensation compared to the younger patients. However, among patients who did meet the Baveno criteria, we did not observe a significant difference in the risk of hepatic decompensation among different age groups. These results underscore the importance of considering age when applying the B7C and B6C. Consequently, we developed a novel clinical pathway that integrates both the Baveno criteria and age, facilitating a more comprehensive evaluation of the risk of hepatic decompensation.

As the WHO declares the advent of a global aging era, there is a growing emphasis on understanding the impact of age on disease prognosis.^17^, ^18^ Although few studies have explored the value of age in predicting hepatic decompensated among patients with compensated cirrhosis, there has been substantial evidence indicating a strong association between age and worse prognosis in patients with chronic liver disease.^14^, ^15^, ^19^ de Jongh FE et al. showed that age was an independent prognostic factor related to survival in chronic hepatitis B.^20^ Similarly, Weissberg JI et al. identified patients aged ≥40 years as having worse outcomes in chronic hepatitis B.^21^ In addition, D'Amico and colleagues reviewed 118 studies and concluded that age was the only variable found to have predictive value for survival that is not part of the Child‐Pugh score and/or its parameters in patients with cirrhosis.^5^ The evidence shows that age needs to be given enough attention in patients with compensated cirrhosis. Albeit with the different cut‐off values of age due to different research purposes in previous studies, the present study found that 60 years old is the best cut‐off value to stratify the risk of hepatic decompensation in compensated cirrhosis.

In the current study, the risk of hepatic decompensation was found to increase with age in patients with compensated cirrhosis. Similarly, a study performed by Lin H et al. found that the relative importance of liver‐related death increases with age in patients with NAFLD.^22^ Furthermore, in patients with NAFLD combined with diabetes, age was able to predict the occurrence of liver‐related events.^23^ The detailed pathophysiological mechanisms of how age is involved in predicting the occurrence of hepatic decompensation are beyond the scope of this paper. However, according to previous reports, aging may lead to an increase in portal blood flow and intrahepatic resistance through complex mechanisms, which drive the development of portal hypertension.^24^, ^25^, ^26^ In this study, we also observed that older patients, compared to younger ones, had significantly higher LSM and proportion of varices. Notably, the risk of other chronic diseases increases in the elderly, such as heart disease, diabetes, and musculoskeletal conditions, and the interaction of these conditions may accelerate the natural course of deterioration in compensated cirrhosis.^12^, ^27^, ^28^

To be more adaptable to clinical practice, we explored for the first time the value of combining the Baveno criteria and age groups for risk stratification of hepatic decompensation. Interestingly, we found that age demonstrated a disparate impact on prognosis in those who met and those who did not meet the Baveno criteria. In patients who did not fulfill the Baveno criteria, the occurrence of hepatic decompensation was significantly higher in the older group than in the younger group. However, in those who met the Baveno criteria, no significant difference in the occurrence of decompensation events between the younger and older groups was observed. The possible reason for this is that liver function was better preserved in the younger group, and the effect of age had a lower impact on prognosis.^25^, ^26^ Additionally, in patients not meeting Baveno criteria, we also observed a higher percentage of varices in older patients compared to younger patients. Yet, no similar differences were observed in patients meeting Baveno criteria. Overall, these results suggested that age might have different effects at different disease stages in patients with compensated cirrhosis.

Based on these results, we constructed a new clinical pathway to divide patients with compensated cirrhosis into low‐, medium‐, and high‐risk groups with significantly different risks of decompensation. First, performing more invasive examinations (e.g., HVPG and endoscopy) is not recommended in all older patients. Because the risk of decompensation is low in the low‐risk group (even in older individuals), HVPG and endoscopy can be avoided in these patients. However, for patients who do not fulfill the Baveno criteria, we recommend that more aggressive measures should be taken if no contraindications exist, regardless of age. It is worth noting that older patients who did not fulfill the Baveno criteria were individually classified into the high‐risk group, as their risk of decompensation was significantly higher. Therefore, for the high‐risk group, a comprehensive diagnostic assessment is necessary, and closer follow‐up should be given even after discharge, as timely detection of portal hypertension and varices in patients is essential to estimate the risk of hepatic decompensation and to guide therapeutic decisions.

The present study has several strengths. First, this is an international multicenter cohort study demonstrating the relationship between age and hepatic decompensation, which makes our results more generalizable. Second, age is a readily available indicator that does not require any clinical examination, nor does it increase the cost of diagnosis and treatment. Therefore, our results are simple and practical that can be applied to routine clinical practice. We acknowledged that there are limitations. First, there are inherent confounding biases due to the retrospective nature of the study design. Second, viral hepatitis is the primary etiology in the present cohort. The applicability of the findings to patients with predominantly other etiologies of cirrhosis remains to be further validated. Finally, HVPG was unavailable in this study.

In conclusion, the current study demonstrated that incorporating age into the Baveno criteria can significantly improve the evaluation of hepatic decompensation risk. Age greater than or equal to 60 years predicts a higher risk of hepatic decompensation in patients with compensated cirrhosis who do not fulfill the Baveno criteria. Therefore, age should be considered before discharging patients with compensated cirrhosis from the surveillance of hepatic decompensation.

## 4 MATERIALS AND METHODS

### 4.1 Study design and participants

This international, multicenter cohort study was initiated by the Liver Health Consortium in China (CHESS). From January 2009 to August 2020, eligible patients were recruited from three centers in China (Tianjin Second People's Hospital, Ruijin Hospital, The Fifth Hospital of Shijiazhuang), one center in Singapore (Changi General Hospital), one center in South Korea (Korea University Ansan Hospital), two centers in Japan (Ehime University Graduate School of Medicine and Hyogo College of Medicine Hospital), and one center in Egypt (Zagazig University Faculty of Medicine). Demographic, routine laboratory, and anthropometric variables were collected from patients. All patients underwent a transient elastography (TE) examination. The inclusion criteria were as follows: (1) age 18 years or older; (2) fulfilled the diagnosis of compensated cirrhosis based on available hospital records, including histological, radiological, and clinical features. Notably, patients were not required to be first diagnosed with compensated cirrhosis. The following patients were excluded: (1) previous hepatic decompensation; (2) hepatocellular carcinoma; (3) previous liver transplantation;(4) portal vein thrombosis; (5) antiplatelet or anticoagulation; (6) alcoholic cirrhosis with significantly ongoing alcohol intake; (7) not receiving primary prophylaxis of the first hepatic decompensation (e.g., nonselective beta‐blockers or endoscopic variceal ligation); (8) non‐sinusoidal portal hypertension; and (9) incomplete follow‐up data. All eligible patients were routinely followed up at 6‐month intervals to monitor hepatic decompensation and death. The present study was authorized by the ethics committee of the First Hospital of Lanzhou University (LDYYLL2020‐19) and complied with the 1975 Declaration of Helsinki. In light of the retrospective nature of the study, the requirement for informed consent was waived.

### 4.2 Transient elastography

Following the manufacturer's guidelines, the LSM was assessed by trained operators utilizing TE with the FibroScan (Echosens). The following results were considered reliable: at least 10 valid acquisitions, IQR less than 30%, and successful rate greater than 60%. The median value represents the patient's final examination results, expressed in kPa. Patients with unreliable results were excluded from the study.

### 4.3 Definition and outcomes

According to the Baveno consensus, meeting B7C is defined as having a PLT ≥ 150 × 10^9^/L and LSM ≤15 kPa; meeting B6C is defined by a PLT > 150 × 10^9^/L and LSM < 20 kPa. Thus, meeting the Baveno criteria is defined as satisfying the B6C and B7C criteria simultaneously. Otherwise, it is considered not to meet the Baveno criteria.

The primary endpoint was the time to the first hepatic decompensation (variceal bleeding, ascites, or hepatic encephalopathy). Patients were censored at the time of hepatic decompensation, death, and the last follow‐up, respectively. To reduce reporting bias, we only incorporated the following objective endpoints in the study: clinically significant ascites that required paracentesis or diuretic therapy, variceal bleeding documented by esophagogastroduodenoscopy, and overt hepatic encephalopathy (defined as west‐haven Grade 2 and beyond) determined by experts.

### 4.4 Statistical analysis

Continuous variables were represented as mean (SD) or median (IQR). Categorical variables were denoted by counts and percentages (%). Qualitative variables were analyzed using either the chi‐square test or Fisher's exact test, whereas quantitative variables were compared through Student's *t*‐test or Mann–Whitney *U*‐test. The median follow‐up time was determined utilizing the reverse Kaplan–Meier method. The restricted cubic spline with four knots (5th, 35th, 65th, and 95th centiles) was used to analyze the relation of age (continuous variable) and HR of first hepatic decompensation by “rms” package.^29^ In addition, we employed Cox proportional hazards regression analyses to examine the relationship between age and the time to hepatic decompensation. For sufficient sample size for analysis, we classified the countries into two groups: China and other countries. In the multivariate analysis, we adjusted sex and center (China and other countries) to reduce the influence of confounding factors. Further, the optimal cut‐off point for age (years), corresponding to the most significant association with hepatic decompensation, was determined by maximally selected rank statistics using “maxstat” for time to the development of hepatic decompensation.^30^, ^31^ In addition, the adjusted probability of free‐hepatic decompensation was calculated with the direct standardization method, and adjusted curves were plotted by “adjustedCurves” package.^32^, ^33^ The probability of decompensation among different risk groups was compared using the long‐rank test. All statistical tests were conducted as two‐sided, with a significance threshold set at *p* < 0.05. Data management and analysis were performed using SPSS 25.0 (IBM) and R V.4.1.3 (R core team) with the additional packages survival, survminer, rms, adjustedCurves, and so on.

## AUTHOR CONTRIBUTIONS

Xiaolong Qi, Chuan Liu, and Shanghao Liu designed the overall study. Shanghao Liu, Jia Li, Yujun Wong, Hyung Joon Yim, Masashi Hirooka, Hirayuki Enomoto, Qing Xie, Erhei Dai, Amr Shaaban Hanafy, Zhujun Cao, Lili Zhao, Kok Ban Teh, Tae Hyung Kim, Young Kul Jung, Yohei Koizumi, Yoichi Hiasa, Takashi Nishimura, Hiroko Iijima, Qingyi Tian, Xinru Guo, and Yansheng Jia collected and curated the data. Shanghao Liu performed statistical analyses, with the supervision and assistance from Jinfang Sun. Chuan Liu and Qingyi Tian accessed and validated the data. Shanghao Liu wrote the original draft. Xiaolong Qi, Chuan Liu, and Yujun Wong reviewed it and edited the manuscript. All authors have read and approved the final manuscript.

## CONFLICT OF INTEREST STATEMENT

All authors declare no conflicts of interest.

## ETHICS STATEMENT

The present study was authorized by the ethics committee of the First Hospital of Lanzhou University (LDYYLL2020‐19) and complied with the 1975 Declaration of Helsinki. The informed consent was waived due to the retrospective nature of the study.

## Supporting information



Supporting Information

## Data Availability

The data of individual deidentified participants will not be shared but are available on request to the corresponding authors.
