# Supplementary material for: Prognostic impact of age on outcomes of hepatic decompensation in patients with compensated cirrhosis (CHESS2102): an international, multicenter cohort study
Source: MedComm (2020). 2024 Nov 3;5(11):e781. doi: 10.1002/mco2.781 (PMC11531654; doi:10.1002/mco2.781)
Supplement: Supplementary file 1 — Supporting Information [file MCO2-5-e781-s001.docx]

**Prognostic impact of age on outcomes of hepatic decompensation in patients with compensated cirrhosis (CHESS2102): an international, multicenter cohort study**

**Authors information:**

Shanghao Liu, Jia Li, Yujun Wong, Hyung Joon Yim, Masashi Hirooka, Hirayuki Enomoto, Qing Xie, Erhei Dai, Amr Shaaban Hanafy, Zhujun Cao, Lili Zhao, Kok Ban Teh, Tae Hyung Kim, Young Kul Jung, Yohei Koizumi, Yoichi Hiasa, Takashi Nishimura, Hiroko Iijima, Qingyi Tian, Xinru Guo, Yansheng Jia, Jinfang Sun, Chuan Liu, Xiaolong Qi

**Table S1: The adjusted hazard ratio between the older and younger groups in China and other countries.**

|  | China (n = 409) | | | Other countries (n = 729) | | |
| --- | --- | --- | --- | --- | --- | --- |
|  | Patients | Events (%) | aHR (95% CI),  p value^*^ | Patients | Events (%) | aHR (95% CI),  p value^*^ |
| Not meeting B7C | 294 | 35 (11.9%) | 2.13 (1.07-4.24),  p = 0.032 | 564 | 78 (13.8%) | 2.56 (1.59-4.12),  p < 0.001 |
| Meeting B7C | 115 | 2 (1.7%) | 7.39 (0.41-134.77),  p = 0.177 | 165 | 3 (1.8%) | 2.98 (0.27-33.0),  p = 0.373 |
| Not meeting B6C | 279 | 35 (12.5%) | 2.06 (1.03-4.12),  p = 0.040 | 513 | 79 (15.4%) | 2.50 (1.56-4.01),  p < 0.001 |
| Meeting B6C | 130 | 2 (1.5%) | 7.31 (0.40-133.89),  p = 0.180 | 216 | 2 (0.9%) | 1.12 (0.07-17.95),  p = 0.937 |

^*^Compare the difference in the occurrence of hepatic decompensated between the younger group (ref) and the older group. Covariates included sex (female [reference] vs. male). The Adjusted hazard ratio was calculated by multivariate COX regression. B7C, Baveno VII criteria; B6C, Baveno VI criteria. aHR, adjusted hazard ratio.


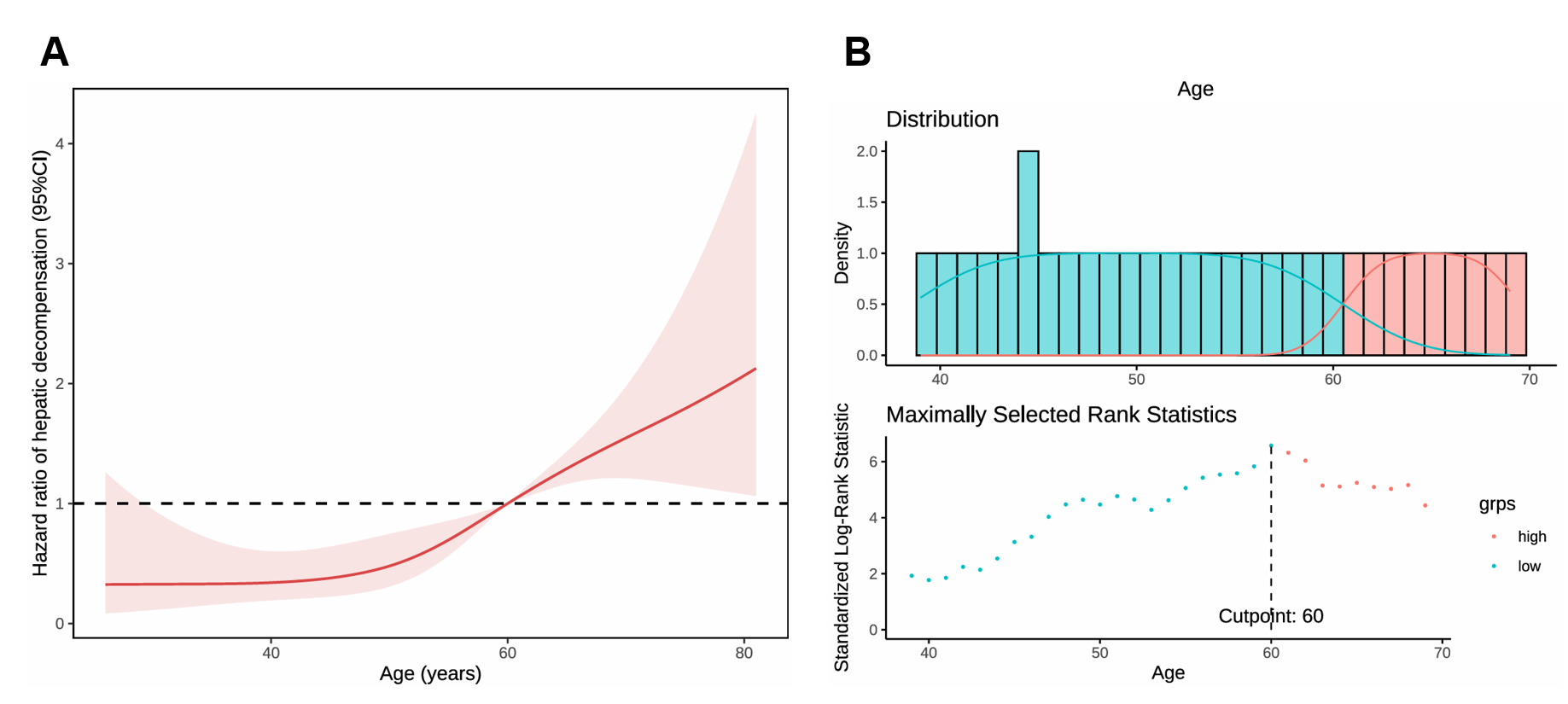


**Figure S1. (A) The association between age (years) and hepatic decompensation in patients with compensated cirrhosis; (B) identifying the optimal threshold of age (years) for hepatic decompensation by maximally selected rank statistics.**
